# Effects of combat training on visuomotor performance in children aged 9 to 12 years - an eye-tracking study

**DOI:** 10.1186/s12887-018-1038-6

**Published:** 2018-02-07

**Authors:** Yan-Ying Ju, Yen-Hsiu Liu, Chih-Hsiu Cheng, Yu-Lung Lee, Shih-Tsung Chang, Chi-Chin Sun, Hsin-Yi Kathy Cheng

**Affiliations:** 1 Department of Adapted Physical Education, National Taiwan Sport University, 250 Wen-Hua 1st Rd, Kwei-Shan, Taoyuan, 333 Taiwan, Republic of China; 2 Office of Physical Education, Chang Gung University, 259 Wen-Hua 1st Rd, Kwei-Shan, Taoyuan, 333 Taiwan, Republic of China; 3 Department of Physical Therapy and Graduate Institute of Rehabilitation Science, College of Medicine, Chang Gung University, 259 Wen-Hua 1st Rd, Kwei-Shan, Taoyuan, 333 Taiwan, Republic of China; 4 Department of Physical Medicine and Rehabilitation, Chang Gung Memorial Hospital, 5 Fu-Hsing Street, Kwei-Shan, Taoyuan, 333 Taiwan, Republic of China; 5 Graduate Institute of Early Intervention, College of Medicine, Chang Gung University, 259 Wen-Hua 1st Rd, Kwei-Shan, Taoyuan, 333 Taiwan, Republic of China; 6 Office of Physical Education, Chung Yuan Christian University, 200 Chung Pei Rd, Chung Li, Taoyuan, 320 Taiwan, Republic of China; 7 Department of Ophthalmology, Chang Gung Memorial Hospital, Keelung, Taiwan, Republic of China; 8 Department of Chinese Medicine, College of Medicine, Chang Gung University, 259 Wen-Hua 1st Rd, Kwei-Shan, Taoyuan, 333 Taiwan, Republic of China

## Abstract

**Background:**

Data on visuomotor performance in combat training and the effects of combat training on visuomotor performance are limited. This study aimed to investigate the effects of a specially designed combat sports (CS) training program on the visuomotor performance levels of children.

**Methods:**

A pre–post comparative design was implemented. A total of 26 students aged 9–12 years underwent 40-min CS training sessions twice a week for 8 weeks during their physical education classes. The CS training program was designed by a karate coach and a motor control specialist. The other 30 students continued their regular activities and were considered as a control group. Each student’s eye movement was monitored using an eye tracker, whereas the motor performance was measured using a target hitting system with a program-controlled microprocessor. The measurements were taken 8 weeks before (baseline), 1 day before (pretest), and 1 week after (posttest) the designated training program. The task used for evaluating these students was hitting or tracking random illuminated targets as rapidly as possible. A two-way analysis of variance [group(2) × time(3)] with repeated measures of time was performed for statistical analysis.

**Results:**

For the children who received combat training, although the eye response improvement was not significant, both the primary and secondary saccade onset latencies were significantly earlier compared to the children without combat training. Both groups of students exhibited improvement in their hit response times during the target hitting tasks.

**Conclusion:**

The current finding supported the notion that sports training efforts essentially enhance visuomotor function in children aged 9–12 years, and combat training facilitates an earlier secondary saccade onset.

## Background

Effective visuomotor coordination is critical for performing functional movements underpinning physical activity and cardiorespiratory fitness in children and adolescents [1]. During the early stages of motor skill development, performance of an unfamiliar task is uncoordinated and requires considerable perceptual and cognitive effort in executing movement [2]. Visuomotor integration begins with the visual perception of objects and events, providing a foundation for cognitive operations [3]. During typical child development, the visual perceptual function expands substantially between the age of 3 and 5 years, becomes stable between the age of 7 and 9 years, and can still improve at the age of approximately 10 years [4]. In addition to strong visual perceptual function, effective and efficient action is required to improve a child’s motor performance.

The principal function of vision is to provide the information required to support an action [5]. Predictive eye movements support the planning and control of goal-directed movements. Both before and during limb movements, visual information is processed and used in the planning, updating and correction of the ongoing movement. For goal-directed movements, various outcome parameters, such as reaction or initiation time, movement time and total response time are associated with each other, and the time spent gives an indication on movement planning, regulation and on-line adaptation [6]. These movement updates and corrections are outcomes from visual feedback processing, and the underlying interactions between the eye and the hand movements can be deduced from eye movement parameters. Recently, it was suggested that such eye movement analyses can be used to differentiate between children with and without developmental coordination disorders [7]. Inherently dynamic movement, such as catching a ball, requires modifying planned movement responses on the basis of visual information about the flight of the ball. For upper limb movement involving finger, elbow and shoulder joints, research has found a clear temporal coupling between primary saccade completion and peak acceleration of the joints [6]. This indicated that visual information guides and controls the goal-directed action [1, 6]. In children with impaired motor coordination, less effective gaze strategies are used in controlled laboratory reaction time tests, visual tracking, and cued reach-to-grasp tasks [8, 9]. Deficits in visuomotor function delay motor performance in children.

A large body of evidence supports the claim that training can improve visual-perceptual function and that sport-specific visual skills can improve sports performance [10–14]. The measurements of response accuracy and response time indicate that, compared to nonexperts, experts fixate on fewer areas of interest for a longer period of time [15]. Differences in visual search behaviors have also been observed, revealing that experts use fewer fixations, fixations of longer duration including longer quiet eye duration, and an earlier onset of the saccade during motor response initiation, compared with nonexperts [16–22]. Causer et al. (2010) conducted an exploratory investigation of the visual search behaviors and gun barrel kinematics used by elite and subelite shooters [16]. The gaze point and gun barrel kinematics were recorded. The elite shooters demonstrated both an earlier onset and a longer relative duration of quiet eye than their subelite counterparts did. Rienhoff et al. (2012) investigated the extent to which quiet eye supports the information pickup of varying fields of vision and determined that differences existed between various skill levels [21]. Harle and Vickers (2001) compared university basketball players who received quiet eye training with two control teams who did not receive similar training [18]. Players who received the training demonstrated improvements in shooting accuracy. Ghasemi et al. (2009) compared the visual skills of expert and novice soccer referees and determined that the expert referees outperformed the novice referees and control group in recognition speed [17]. The aforementioned results indicate that sport-specific visual skills can be improved through specific training programs.

When a performer produces eye movements to bring objects into the foveal field, a saccade (a rapid movement of the eye) reduces visual sensitivity, thus hindering the information processing [22]. A saccade reflects a critical period of cognitive processing when motor skill parameters are programmed. Expert performers use proficient visual search strategies in various sports [16–22]. Among the various sports, combat sports (CS) and ball activities place greater emphasis on accurate object tracking and swift responses [23–28], particularly for CS such as karate, taekwondo, and judo. These activities require the eye of the athlete to stabilize and fixate on indeterminate target locations prior to an action. Because CS training emphasizes accurately detecting visual cues and generating effective motor responses in combat environments [29], the contextual information from CS training may be retained, and the abilities may be transferred to another similar context.

Current studies on CS training have concluded that instead of processing information related to the shapes of opponents’ faces, hands, legs, and outfits, the training of elite karate and boxing athletes focuses on the skillful, rapid processing of visuospatial and motor information when performing combat moves [23, 30, 31]. Babiloni et al. (2010) measured electroencephalographic changes in karate athletes; those measurements revealed that elite athletes were characterized by reduced cortical activation in their dorsal cortical visual pathways during movement execution [23]. Ripoll et al. (1995) used a video-oculography apparatus and reported that although the reaction times of expert athletes did not differ from those of novice athletes, expert athletes demonstrated minimal movement and chunked the different items present in the visual field [30]. These studies indicate that expert athletes demonstrate neural efficiency when engaging in social cognition.

Since the information concerning the effects of combat training intervention on visuomotor performance is scarce, particularly regarding children at the visual perceptual development stage, the current study aimed to provide children with a specially designed CS training program and to compare the effects of the program with those of the general sports (GS) training program on visuomotor performance in another similar context. The hypothesis of this study was that children who receive the CS training program outperform children who receive the GS training program in terms of visuomotor performance in a target hitting task.

## Methods

### Participants

Convenience sampling was used to recruit participants. This study included two classes of children aged 9–12 years from a local primary school. The inclusion criteria were a visual acuity of at least 5/6 (Snellen’s E chart) and no upper extremity sensorimotor deficits [32]. Children who participated in sports teams (e.g., baseball and karate) and those who could not attend every training session were excluded. The two classes included students with similar characteristics (e.g., gender, age, and height). The study protocol was reviewed and approved by the Ethics Committee at Chang Gung Memorial Hospital (No. 101-3718B). Written informed consents were obtained from guardians on behalf of the enrolled children.

### Experimental protocol

CS training was provided to one class during physical education classes. The other class received a regular physical education routine to serve as a control. For 8 weeks, 40-min training sessions were conducted twice a week. The CS training program was designed by a karate coach, who has 20 years of combat experience and was a national karate athlete, and a motor control specialist, who is a university professor with 20 years of teaching and clinical experience [25, 29, 33, 34]. The intervention, delivered in a group setting, was structured as follows: (a) a 16-min warm up, including circular movements of the neck, shoulders, hips, and other joints of the extremities, and a march in place; (b) 20-min combat-specific skills including one-on-one attacks, counterattacks, defense, and attack combinations (mainly upper-limb and lower-limb karate techniques such as “punch with forward anterior hand,” “punch to the head,” and “circular kick to the body”, along with training that focused on perceiving an opponent or object before a response; and (c) a 4-min cool down. These programs were intended to improve eye, hand, and foot coordination including agility and reaction speed. The entire lesson was led by a professional karate teacher. The in-class karate teacher was unaware of the purpose of the study. The total intervention time was 40 min each for both groups. The other class continued the activity routines to serve as the control group.

The performance of these students was evaluated using two events. The events required the participants to hit (the motor event) or visually track (the visual event) random illuminated targets as rapidly as possible. The motor event was monitored with a target hitting system that used a program-controlled microprocessor. The visual event was monitored using an eye tracker with a simulated hitting test. Measurements were taken at three specific time points: 8 weeks before the training (baseline), 1 day before the training (pretest), and 1 week after the training (posttest). Data collected 8 weeks before training served as baseline.

### Variables and equipment

#### The motor event

**Fig. 1:**
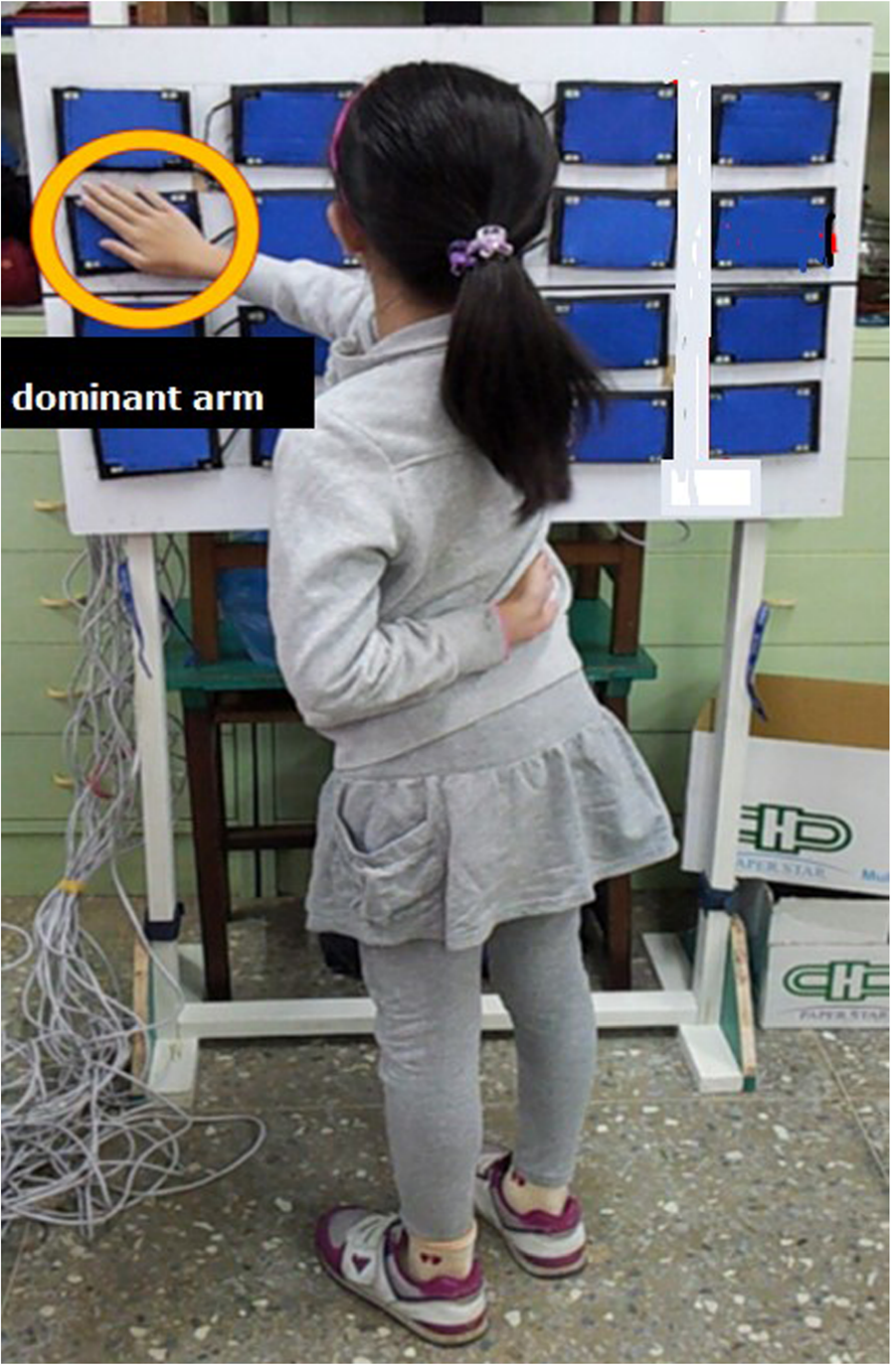
Picture of a child performing the target movement. The front panel is embedded with program-controlled microprocessor

**Fig. 2:**
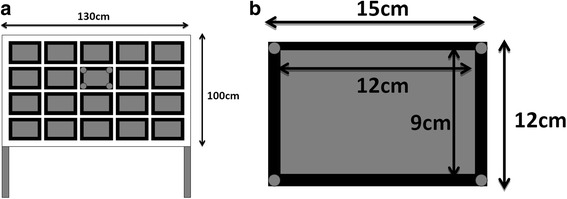
**a** The standing frame and the targets of the hitting system. **b** Each target contained embedded sensors and four red light-emitting diodes (LEDs) in the target corners

A self-designed target hitting system with an embedded program-controlled microprocessor (Fig. 1) was used to measure the participants’ motor performance. It comprised a standing frame with 20 hitting targets (a 0.15 m × 0.12 m blue rectangle; Fig. 2). Each target contained embedded sensors and four red light-emitting diodes (LEDs) in the target corners; the lighting sequence was managed through a program-controlled microprocessor (LabVIEW 8.0, National Instruments, Austin, TX). The task was to hit the target that lit up as quickly as possible. This task was used to evaluate hand–eye coordination. Before the testing began, participants stood in front of the hitting system with their feet shoulder-width apart and one arm-length between the student and the target. The middle of the frame was set to the participants’ eye level.

**Fig. 3:**
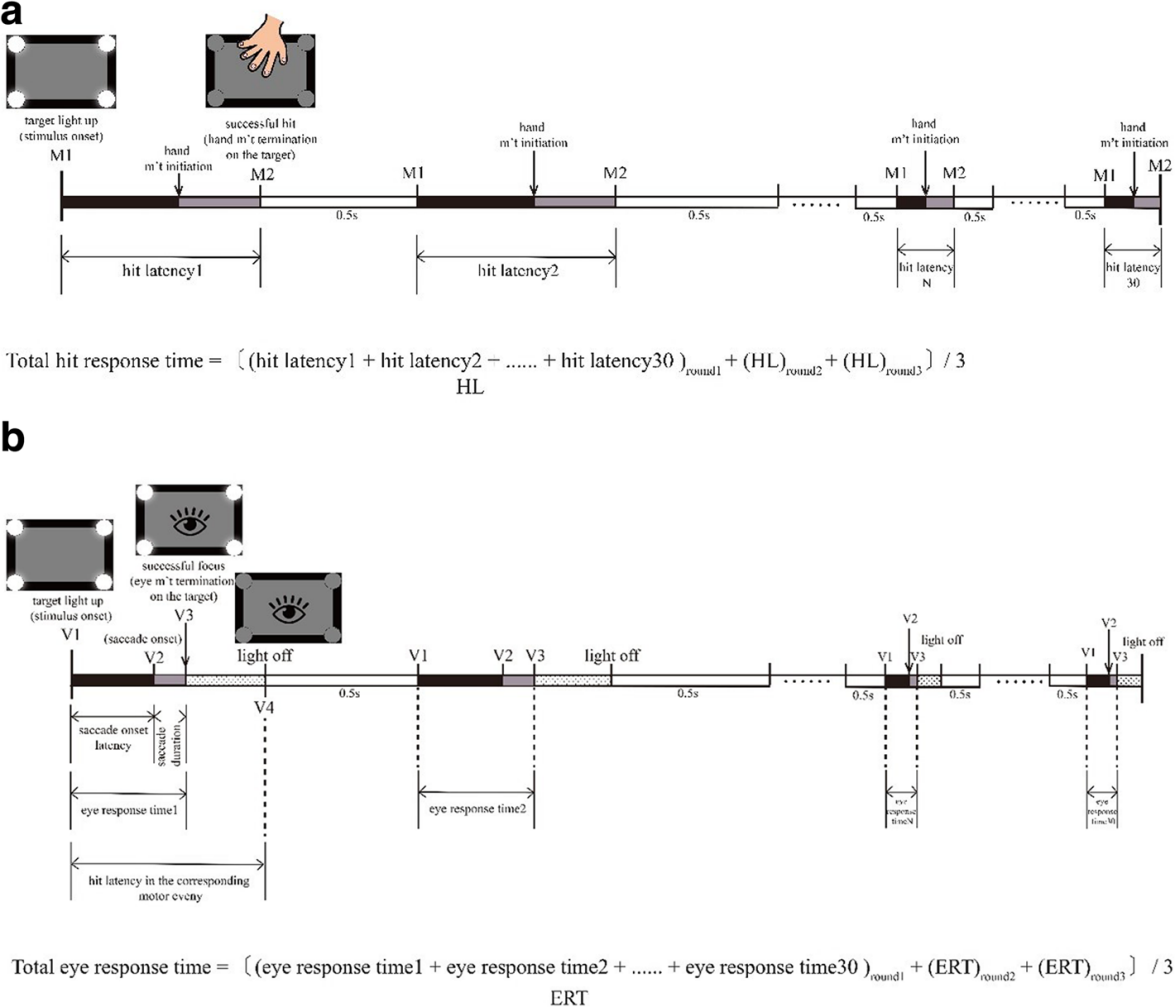
The timing of the motor (**a**) and the visual (**b**) events

A signal was made indicating the start of the test, and participants were requested to place their non-dominant hands behind their backs. Two to five seconds after the ready signal, one of the targets would light up (stimulus onset, M1). The participants were instructed to hit the illuminated target as rapidly as possible by using their dominant hand. Only one target lit up at a time, and the target did not go dark until the participant had successfully hit it (hand movement termination on the target, M2). The next target would light up 0.5 s later. If a hit did not trigger the pressure sensor, the LED would stay on until the pressure was registered. The program controlled which LED would go first and then succeeded by three preprogrammed random sequences. Each participant received three rounds of target hitting, and each round involved 30 illuminated targets. Participants took a 5-min break after each round. The complete test lasted between 10 and 15 min. The “hit latency” and the duration between M2 and M1 were recorded for each round. The “total hit response time” of each round was the sum of the hit latency values recorded for 30 successful hits. For each participant, the “total hit response time” in the three rounds were averaged to derive the participant’s motor performance (Fig. 3a).

#### The visual event

**Fig. 4:**
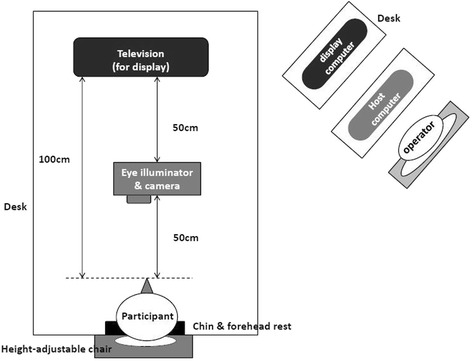
The experimental setup for the eye protocol. The layout represents the relative position of the devices and participants

An eye tracker (EyeLink 1000, SR Research Ltd., ON, Canada) was used to measure the onset latency and duration of the saccade for the simulated hitting. The eye tracker comprised a program-driven host personal computer (PC), a display PC with a 65" screen (Viewsonic, TW) to display the eye tracking pattern, an eye illuminator to indicate the pupil (infrared wavelength 890 nm), and a camera to detect pupil position (monitoring frequency 1000 Hz). The analysis was conducted using the dominant eye of participants, which was determined using Dolman’s “hole-in-card” test. Participants sat in a height-adjustable chair 1 m in front of the display screen, set their chins on the chin rest, and kept both feet flat on the floor with their eyes level with the middle of the screen. According to the user manual of the eye tracker, the distance between the screen and participant was determined on the basis of screen size. The chin rest was necessary to avoid head movement during pupil detection. The illuminator and camera were placed on a desk halfway between the eyes and the display screen (Fig. 4). The operator controlled the eye tracker by using the host PC, and the display PC displayed the preprogrammed tracking sequence. After participants were in a comfortable sitting position, the system was set up and calibrated for the specific participant. Subsequently, the participants completed a three-round simulated hitting test with each round comprising 30 illuminated targets. A 5-min rest was provided after each round. The complete test lasted between 10 and 15 min. The tracking pattern and light on–off time were programmed on the basis of the aforementioned target hitting system. The screen layout mimicked the layout of the target hitting system. The layout of the system was proportional to the display screen size to ensure the same visual angles while evaluating both movements (max. horizontal/vertical visual angle: 68°/46°). Each corner of the blue rectangle has a red light, which would light up and turn off in the same lighting sequence as that of the target hitting system. The turn-off time of the light was controlled using a custom-written program (MATLAB; The MathWorks, Natick, MA, USA).

The participants were instructed to focus on the illuminated target as quickly as possible. Only one target was lit up at a specific time. For each round, the system registered the time each target lit up (stimulus onset, V1), the time of the saccade onset (V2), and the time the participant eye movement termination on the target (V3). The time-point (V4) at which the lights went off in the visual event was programmed to be equal to the “lights off time” in the motor event. Then the next target would light up 0.5 s later. The primary saccade onset was identified using a custom-written program with a velocity threshold of 20°/s, whereas the velocity threshold for secondary saccade onset was 15°/s. Primary saccades were rejected if their latency was > 500 ms or peak velocity was < 100°/s. These exclusion criteria are commonly used in saccade studies to exclude trials with delayed saccade. The following variables were then calculated: the “saccade onset latency” for the primary saccade was the duration between V2 and V1; the “saccade duration” was the duration between V3 and V2; and the sum of the “saccade onset latency” and the “saccade duration” was the “eye response time.” The total “eye response time” for the three rounds was averaged to derive the eye performance of participants (Fig. 3b). The onset latency for the secondary saccade (the corrective saccade that follows the primary one)was also examined. It was defined as the interval from the end of the primary saccade to the initiation of the next saccadic movement. Secondary saccades were rejected if their latency was > 350 ms.

Data were analyzed using SPSS software (Version 17.0, SPSS Inc., Chicago, IL, USA). Descriptive statistics were used to calculate the participants’ demographics. Two-way analysis of variance [group(2) × time(3)] with repeated measures on time was performed for the total hit response time, the eye response time for simulated hitting, and the saccade onset latency to evaluate the effects of the 8-week training intervention on the participants’ visuomotor performance. The significance level was set to .05. Least significant differences were used for multiple comparisons whenever necessary.

## Results

A total of 65 children were recruited. Nine of them were excluded because they could not participate in all training sessions or they had already participated in other regular sports activities. Data from the remaining participants (56 children) were collected and analyzed; 30 participants were assigned to the CS group and 26 were assigned to the control group. For ethical reasons, the nine excluded children still received the training sessions, but their data were not collected. Table 1 shows the background demographics of the participants. No significant differences were observed.

**Table 1:** Background demographics of the participants (*n* = 56)

	CS group (Mean ± SD)	GS group (Mean ± SD)	df	t	Group differences (*p* value)
Number of participants	30	26			
Gender (male/female)	12/18	11/15	54	0.172	0.864
Age (years old)	9.0 ± 0.6	9.0 ± 0.6	54	−0.424	0.673
Height (cm)	131.0 ± 6.8	132.4 ± 6.1	54	0.787	0.435
Weight (kg)	31.5 ± 9.0	30.4 ± 8.1	54	−0.470	0.640
Visual acuity (left eye)	1.1 ± 0.2	1.0 ± 0.3	54	−1.229	0.225
Visual acuity (right eye)	1.1 ± 0.2	1.0 ± 0.3	54	−1.629	0.109

Group: *CS* Combat sport, *GS* general sport

**Fig. 5:**
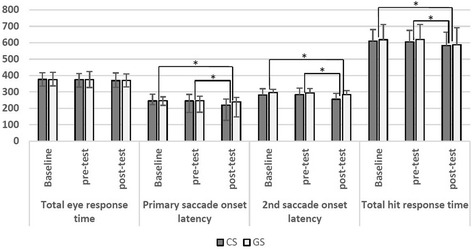
Results for total eye response time, saccade onset latency (primary and secondary), and total hit response time in two groups (unit: ms). CS: combat sports, GS: general sports

For the CS group, the baseline, pretest, and posttest eye response times were 376.3 ± 40.3, 374.3 ± 37.8, and 368.9 ± 46.7 ms, respectively. For the control group, the baseline, pretest, and posttest the eye response times were 374.6 ± 45.2, 375.4 ± 48.5, and 370.3 ± 39.1 ms, respectively. Statistical analyses revealed no significant differences in time (*p* = 0.306, η^2^_p_ = 0.019), in group (*p* = 0.977, η^2^_p_ = 0.000), or the interaction (*p* = 0.785, η^2^_p_ = 0.001; Fig. 5).

For the CS group, the baseline, pretest, and posttest onset latencies of the primary saccade were 244.9 ± 40.5, 245.4 ± 39.5, and 219.7 ± 36.0 ms, respectively. For the control group, the baseline, pretest, and posttest onset latencies were 246.7 ± 23.7, 246.3 ± 28.3, and 239.2 ± 27.9 ms, respectively. The statistical analyses revealed significant differences in time (*p* < 0.001, η^2^_p_ = 0.221), and interaction (*p* = 0.034, η^2^_p_ = 0.080), but not in group (*p* = 0.365, η^2^_p_ = 0.015; Fig. 5). Multiple comparisons indicated that time differences existed between the baseline and posttest (*p* < 0.000, 95% CI = 8.224–24.394) as well as between the pretest and posttest (*p* < 0.000, 95% CI = 8.851–23.885). However, no time difference was observed between the baseline and pretest (*p* = 0.978). The control group had a similar primary saccade onset latency for all three time periods, whereas the CS group exhibited a significant decrease in their saccade onset latencies from the pretest to the posttest. In the posttest, the saccade onset latency in the CS group was significantly shorter than that in the control group (*p* = 0.027, 95% CI = 2.338–36.681).

For the CS group, the baseline, pretest, and posttest onset latencies of the secondary saccades were 280.6 ± 39.2, 283.6 ± 38.9, and 255.5 ± 36.0 ms, respectively. For the control group, the baseline, pretest, and posttest onset latencies were 296.7 ± 19.5, 294.0 ± 26.7, and 283.3 ± 25.9 ms, respectively. The statistical analyses indicated significant differences in group (*p* = 0.024, η2 _*p*_ = 0.090), time (*p* = 0.000, η2 _*p*_ = 0.300), and interaction (*p* = 0.041, η2_p_ = 0.064) (see Fig. 5). Multiple comparisons indicated that differences in time existed between the baseline and posttest (*p* = 0.000, 95% CI = 11.688–26.750) as well as between the pretest and posttest (*p* = 0.000, 95% CI = 12.033–26.713). However, time differences were not observed between the baseline and pretest (*p* = 0.943). The control group had similar primary saccade onset latencies at all three time periods, whereas the CS group exhibited a significant decrease in their saccade onset latencies from the pretest to the posttest. In the posttest, the saccade onset latency in the CS group was significantly shorter than that in the control group (*p* < 0.002, 95% CI = 11.127–44.490).

For the CS group, the baseline, pretest, and posttest total hit response times were 610.2 ± 69.9, 605.0 ± 70.4, and 583.4 ± 81.8 ms, respectively. For the control group, the baseline, pretest, and posttest values were 618.6 ± 93.0, 619.3 ± 91.6, and 587.9 ± 104.4 ms, respectively. The statistical analyses indicated a significant difference in time (*p* < 0.000, η^2^_p_ = 0.207) but not in group (*p* = 0.678, η^2^_p_ = 0.003) or interaction (*p* = 0.563, η^2^_p_ = 0.006; Fig. 5). Multiple comparisons indicated that time differences existed between the baseline and posttest (*p* < 0.000) as well as between the pretest and posttest (*p* = 0.001). However, the time differences were not observed between the baseline and pretest (*p* = 0.297).

## Discussion

Over the years, empirical studies on the correlation between visual ability and sports performance have supported the critical role of visual attention in expert athletes [10, 15, 17, 20, 23]. Visual information is crucial in guiding goal-directed movements. A previous research showed that the eyes tend to arrive at the target before the hand starts to move [35]. When vision is not directed towards the target, the accuracy of the processing movement is reduced. A foveal fixation on the target allows the precise monitoring of target movements relative to the hands.

The current study investigated whether children who received combat-specific training outperform children who received general sports training in a target hitting task in terms of visuomotor performance. The results can be discussed under two aspects: visual and motor. For the visual aspect, no significant differences were found in group, time, or interaction before and after the training intervention for the total eye response time. However, both types of training shortened the primary and secondary saccade onset latencies, as indicated by the significant statistical interaction with group × time. In the posttest, the saccade onset latency in the CS group was significantly shorter than that in the control group, indicating that combat training can facilitate earlier primary and secondary saccade onsets. Gaze control is trainable. Moreover, skilled performers learn to use their gaze efficiently; thus, they know where and when to fixate their gaze while executing the motor skill [7]. This attention process is defined as the cognitive system that facilitates the selection of necessary information from the environment and inhibits other information from further processing [36]. According to Babiloni et al. (2010), training of elite karate athletes emphasizes on the skillful rapid processing of visuospatial and motor information during karate actions [23]. Mori et al. (2002) also stated that competitive high-level sports, including CS, are characterized by severe spatial and temporal constraints imposed on the performer by regulations and the opponents [25]. Hence, an effective performance requires the efficient execution of motor behavior in addition to a high level of perceptual ability. Therefore, the CS training includes aspects that are required to facilitate the saccade reaction.

For the second aspect, the total hit response time significantly decreased (*p* < .000) after intervention. However, the total hit response time of the two groups did not differ significantly (*p* = .678). These results indicated that both groups exhibited improvement in the time required to complete the target hitting action. The hitting motion can be decomposed by dividing the hit latency into a reaction period (before “hand movement initiation”) and an arm movement period (between “hand movement initiation” and “hand movement termination on the target”). Previous motor control studies investigating CS have reported that movement time was a primary determinant of the total response time of the attack [34, 37]. Because the eye response time in the current study did not reveal any significant change, the significant changes in the total hit response time after training were primarily attributable to the movement of the arm during the program. Because CS training improved the general visuomotor performance, future research can use more visuomotor tests to measure the changes.

Although no direct link or evidence can be draw between this study and the functional impact on combat sports activities, several researchers have investigated the functional significance of eye movement strategy and its association with skills in other sports, such as batting. Land and McLeod (2000) found a systematic relationship between gaze and batting skill [38]. Batters were found to track the ball for a short period after ball-release before making a predictive saccade to anticipate where the ball would bounce. Critically, the predictive saccade occurred earlier as the skill level of the batter increased, reflecting a superior ability to predict the future landing point of the ball. This highlights that there may be some functional advantage in producing a predictive saccade when hitting a moving target. In this study, combat sports can also take advantage of predictive saccade since hitting a moving target or evading an enemy’s attack relys heavily on the predictive function.

For children with low motor coordination, gaze registration techniques might provide insight into how external visual information can be used to guide and control goal-directed motor actions [1, 39]. The CS training used in this study particularly shortened the saccade onset latency and is thus an efficient technique to improve gaze registration. Because CS training emphasizes on accurately detecting visual cues and generating effective motor responses in combat environments [29], the contextual information from CS training may be retained, and the abilities may be transferred to another similar context. The results can be used to assist children with special needs, such as those with developmental coordination disorders (DCD) or low motor coordination. Because children with DCD and typically developed children were equally capable of using a prelearned visuomotor transformation to adapt to the new parametric features of a task [1, 6], subjecting the children with DCD to specific training might improve their movement performance.

The current study has some limitations. First, because all school-age children participate in regular physical education classes, the control group was free from CS but not from general physical activities. However, we detected any change that was simply a function of time and general development by monitoring the performance of children subjected to these regular class activities before the CS intervention. Second, no retention test was performed because the semester was approaching an end after the 8-week course. During summer vacation, it was difficult to control the physical activities of the participants; therefore, we were unable to perform a retention test for group comparison. Third, the vision and motor performance data were collected using two separate tests due to technical difficulties. To ensure that the camera detected precise pupil position, the participants were required to set their chins on a chin rest to keep their head stable. Technically, combining the target hitting test with the current eye tracking method was not feasible. Numerous efforts were made to ensure that the eye response captured by the eye tracker replicated that during the target hitting movement. The tracking pattern and timing of the illuminated targets displayed on the TV screen were programmed based on the aforementioned target hitting system. The screen layout was a proportional minimization of the target hitting system in reference to the size of the display screen and the distances of the participants.

## Conclusion

The current finding supported that sports training can enhance the visuomotor function in children aged 9–12 years. It indicated that although the control group exhibited a similar saccade onset latency across all the three time points, the CS group exhibited a significant decrease in their saccade onset latencies from the pretest to the posttest. In the posttest, both types of training mitigated the saccade onset latency; however, the combat-specific training demonstrated superior improvements. Moreover, both groups showed improvement in the time required for the target hitting action. These results can be used to assist children with special needs, such as those with DCD or low motor coordination in training their gaze behavior including the efficiency of gaze registration.

## Abbreviations

CS: Combat sports
DCD: Developmental coordination disorders
GS: General sports
